# The difficulty of the postural control task affects multi-muscle control during quiet standing

**DOI:** 10.1007/s00221-016-4602-z

**Published:** 2016-03-04

**Authors:** X. García-Massó, M. Pellicer-Chenoll, L. M. Gonzalez, J. L. Toca-Herrera

**Affiliations:** Department of Teaching Music, Arts and Body Expression, University of Valencia, Valencia, Spain; Department of Physical Education and Sport, University of Valencia, Valencia, Spain; Department of Nanobiotechnology, Institute for Biophysics, University of Natural Resources and Life Sciences Vienna (BOKU), Muthgasse 11, 1190 Vienna, Austria

**Keywords:** Electromyography, Coherence, Synergy, Quiet standing

## Abstract

The aim of this study was to compare the electromyographic (EMG) coherence between the lower limb and the core muscles when carrying out two postural tasks at different difficulty levels. EMG was recorded in 20 healthy male subjects while performing two independent quiet standing tasks. The first one involved a bipedal stance with the eyes open, while the second consisted of a dominant unipedal stance also with the eyes open. The obtained EMG signals were analysed by computing estimations of EMG–EMG coherence between muscle pairs, both singly (single-pair estimations) and combined (pooled estimations). Pooled and single coherence of anterior, posterior, core, antagonist and mixed pairs of muscles were significant in the 0–5 Hz frequency band. The results indicate that core and antagonist muscle groups, such as the anterior and posterior muscles, share low-frequency neural inputs (0–5 Hz) which could be responsible of the M-modes assembly. The core muscles could therefore provide the necessary synergy to maintain spine stability during the balancing exercise. Finally, differences in EMG–EMG coherence suggest that the muscle synergies formed during unipedal stance tasks are different from those established during bipedal stance.

**Electronic supplementary material:**

The online version of this article (doi:10.1007/s00221-016-4602-z) contains supplementary material, which is available to authorized users.

## Introduction

From a mechanical point of view, human vertical posture is an unstable system that needs control mechanisms to maintain the centre of gravity inside the support base (Suzuki et al. 2012). Even though several studies have focused on modelling quiet stance control obtaining important information about its mechanism, the system of study was reduced to either an inverted or a double-inverted pendulum (Masani et al. 2003; Asai et al. 2009; Gawthrop et al. 2011; Suzuki et al. 2012). However, experimental data show that a great number of joints and muscles are involved in quiet standing balance, resulting in redundant degrees of freedom (Günther et al. 2009, 2011). Bernstein (1967) postulated that the central nervous system (CNS) simplifies the redundant degrees of freedom by activating synergies (Shumway-Cook and Woollacott 2007). These synergies are formed by groups of muscles that act together to perform the same function.

On this question, Krishnamoorthy et al. (2003) determined the existence of three synergistic muscle groups (known as muscle modes or M-modes) that maintain the balance in an upright stance using the uncontrolled manifold (UCM) hypothesis. The first group is composed by the muscles of the posterior area (e.g. gastrocnemius and biceps femoris), the second one by the anterior muscles (e.g. tibialis anterior and rectus femoris) and the last one by the rectus abdominis. This clustering was obtained taking into account the effects of these muscles in CoP displacement: the anterior muscles are used to move CoP forwards, while the posterior muscles displace it backwards. Another study found M-modes co-contraction during load release tasks in unstable upright posture (Krishnamoorthy et al. 2004). These M-modes were presented at calf (tibialis anterior and triceps surae), thigh (vastus lateralis, rectus femoris and biceps femoris) and core muscles (rectus abdominis and erector spinae), and appeared as a consequence of the high task difficulty (Krishnamoorthy et al. 2004). Overall, in this study the authors determined the existence of five different M-modes (i.e. two reciprocal and three co-contractions). Moreover, the authors supported the hypothesis that the CNS chooses only three of these M-modes to control the posture during upright stance. This choice can depend of factors such as task difficulty or expertise.

Furthermore, it was demonstrated that anterior and posterior M-modes are correlated neuronal inputs (Danna-Dos-Santos et al. 2014, 2015). This was figured out from electromyographic (EMG) coherence analysis between the muscles that form the M-modes during bipedal quiet stance. Thus, the mechanism responsible for the synergic action could be attributed to these correlated inputs that the CNS sends to the muscles.

The effect of visual information in the EMG–EMG coherence of anterior and posterior M-modes has also been studied (Danna-Dos-Santos et al. 2015). The authors observed that visual information plays a key role in the generation of common inputs to the muscles. In the same work, the authors postulated that it might be relevant to perform studies taking into account more muscle groups and conditions, in order to gain understanding of the CNS role and muscle synergies in balance control.

Moreover, during daily and sport activities single-leg stance periods are performed. This postural configuration has been considered to be more complex than bipedal stance from the point of view of postural control (Vuillerme et al. 2001; Paillard et al. 2006). In addition, several studies have focused on the effect of training (Paillard et al. 2006), fatigue (Bizid et al. 2009; Bisson et al. 2011) or foot type (Hertel et al. 2002) in unipedal stability. Nevertheless, there are few studies that confirm muscle coordination or muscle synergies during unipedal stance test. Danna-dos-Santos and co-workers found the anterior and posterior M-modes during unipedal stance task in which voluntary sway in anteroposterior direction was performed (Danna-Dos-Santos et al. 2008). Nevertheless, M-modes co-contraction was not found although the task difficulty.

The primary aim of the present study was to analyse the EMG–EMG coherence between the muscles that forms reciprocal (i.e. anterior and posterior) and co-contraction (i.e. at calf, thigh and core) M-modes. Our second aim was to determine the effect of the task difficulty on muscle coherence by comparing EMG–EMG coherence between bipedal and unipedal stance conditions.

## Methods

### Participants

Prior to subject’s recruitment, a sample size calculation was performed using G*Power 3.1 (University of Düsseldorf, Düsseldorf, Germany). Based on the data published in Danna-Dos-Santos et al. (2015), a sample size of 16 was necessary to detect an effect size of 0.92 when Wilcoxon signed-rank test was performed. This effect size was obtained during comparisons between bipedal eyes open and bipedal eyes closed conditions. We assume that effect size equal or higher than 0.92 will be found between bipedal and unipedal conditions. Therefore, 20 subjects were recruited to participate in the study (four more due to possible loss of data).

The average (SD) age, weight and height were 23.3 (4.52) years, 78.2 (9.84) kg and 1.78 (0.07) m, respectively. The participants had no history of neurological or muscular disorders. Eighteen subjects were right-footed and two left-footed, based on their kicking preference. Informed consent was obtained from all individual participants included in the study. The research project was approved by the University Institutional Review Board, and the procedures were in accordance with the Declaration of Helsinki.

### Experimental procedure

**Fig. 1:**
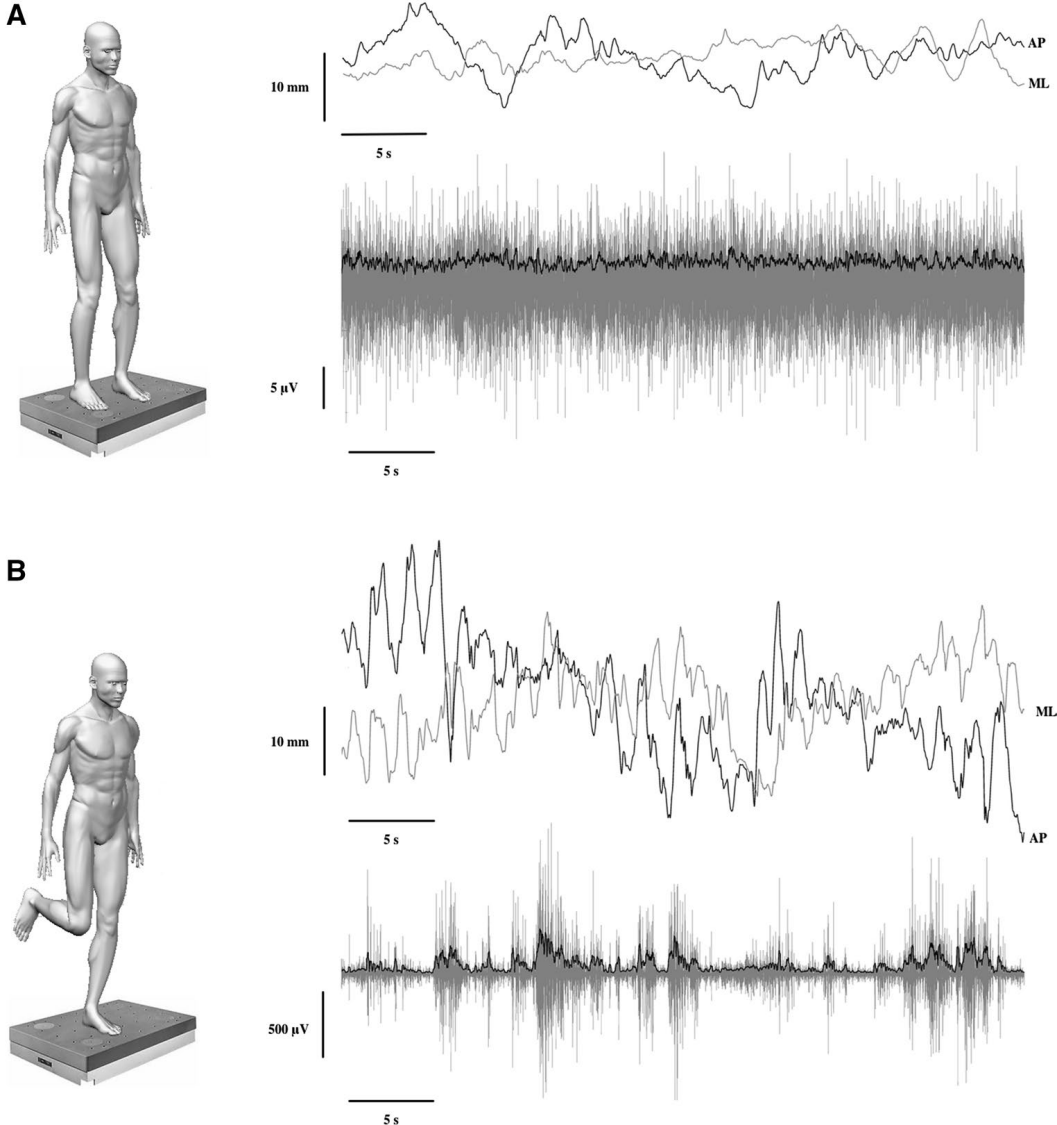
Experimental conditions and examples of tibialis anterior electromyography and centre of pressures signal. In **a**, the centre of pressure displacement and the electromyographic activity of tibialis anterior during bipedal stance condition are shown. *Up*, the centre of pressure displacement in anteroposterior (AP; in *black*) and mediolateral (ML; in *grey*) is indicated. *Down*, the raw electromyographic signal of tibialis anterior is plotted in* grey*, while the root mean square is plotted in black. In **b**, the centre of pressure displacement and the electromyographic activity of tibialis anterior during unipedal stance condition are shown. *Up*, the centre of pressure displacement for anteroposterior (AP; in *black*) and mediolateral (ML; in *grey*) is shown. *Down*, the raw electromyographic signal of tibialis anterior is plotted in grey, while the root mean square is marked in* black*

All the participants performed two independent quiet standing trials. One involved a bipedal stance with the eyes open, and the other consisted of a dominant unipedal stance, also with the eyes open (Fig. 1). The order was randomized to avoid any influence of this factor on the results. Each trial had duration of 50 s with a rest period of 60 s between trials. In the bipedal stance, the subjects were barefoot and still in a relaxed manner with their arms by their sides. Each subject adopted the same foot placement (i.e. heels separated by the width of the shoulders and toes pointing forward). In the unipedal stance, the subjects stood barefoot on their dominant foot. The knee of the non-supporting leg was flexed 90° to ensure that it did not make contact with the floor during the entire trial. If the subjects bent their trunk or arms, as well as they touched down with the non-supporting limb, the trial was discarded. Therefore, after a recovery period of 60 s the participants performed another trial. This procedure was repeated until one successful trial could be recorded. A point of reference (5 cm in diameter) was placed in front of the subject at eye level at a distance of 2 m for each trial. All the subjects were informed of the importance of maintaining these postures and were asked to stand as still as possible.

### Centre of pressure measurements

A 400 mm × 600 mm × 45 mm portable force plate (9253-B11, Kistler Instrument AG, Winterthur, Switzerland) was used to acquire the centre of pressure displacements. The platform was placed on a stable surface on the floor to avoid distortion and noise in the signal. The force plate contained four piezoelectric sensors and each recorded the force produced in three spatial directions. The forces that exerted along the *x-*, *y-* and *z*-axis were recorded at a frequency of 1 kHz, amplified (Kistler amplifier) and converted A/D using a 16-bit card (CIODAS 1600). The centre of pressure (CoP) displacement in anteroposterior (AP) and mediolateral (ML) directions was obtained using the manufacturer’s analysis software (Bioware, Kistler Instrument AG, Winterthur, Switzerland).

### Electromyography measurements

To acquire the surface electromyographic signals produced during the trials, we used a ME6000 biomonitor (Mega Electronics, Ltd., Kuopio, Finland). Prior placing the electrodes, the skin was prepared by shaving the area and also cleaning it with alcohol in order to reduce impedance as much as possible. Pre-gelled bipolar surface electrodes Ag/AgCl (Blue sensor M-00-S; Medicotest, Ølstykke, Denmark) were placed at an interelectrode distance of 20 mm on the following muscle groups: 1. gastrocnemius medialis (GM), 2. tibialis anterior (TA), 3. vastus medialis (VM), 4. biceps femoris (BF), 5. rectus abdominis (RA), 6. external oblique (EO) and 7. erector spinae (ES). The reference electrode was placed between the active electrodes at approximately 10 cm from each one, as per the manufacturer’s specifications. The electrodes were placed according to the SENIAM recommendations (www.seniam.org) on the dominant side of the body.

All signals were acquired at a sampling frequency of 1 kHz, amplified and converted analogue/digital. All records of myoelectrical activity (µV) were stored on hard drive for later analysis. A trigger was used to synchronize CoP and EMG measurements.

### Data processing

The data analysis was performed with MATLAB R2013a (Mathworks Inc, Natick, USA). CoP signals were low-pass dual-pass filtered using a second-order Butterworth IIR filter with a cut-off frequency of 15 Hz. The first 10 s were then cut out before the computation of the variables. In the time domain, the balance variables computed were the 95 % confidence ellipse area (EA), mean velocity (MV) in anteroposterior (AP) and mediolateral (ML) direction and root mean square (RMS) amplitude in AP and ML direction. Frequency domain analysis was also performed. The power spectral density was estimated using a fast Fourier transformation (periodogram function in MATLAB) using a Hanning window of 2^10^ data points. Finally, the median frequency and the 80 % energy frequency (80 % of spectral energy is below this frequency) were calculated.

EMG signals were pre-processed to delete noise and interference. First, we applied a band-pass second-order Butterworth IIR filter with 20–400 cut-off frequencies in direct and reverse directions. Independent component analysis was used to cancel out electrocardiogram interference in the trunk muscles (i.e. RA, ES and EO) (Mak et al. 2010). This was of great importance because the presence of electrocardiogram signals in EMG could increase the coherence level between core muscles (Grosse et al. 2002).

The first 10 s of the signals were discarded in order to use the same time period in the CoP and EMG signals. In the time domain, a RMS moving window of 100 ms was applied. The mean value of the RMS signal was then computed as a variable of the magnitude of muscle activation.

In the frequency domain, EMG signals were analysed by estimating the EMG–EMG coherence between muscle pairs (single-pair estimations) separately and combined (pooled estimations). Coherence analysis was performed using the full-wave rectified EMG signal. Rectification was performed following the recommendations established in computational studies (Boonstra and Breakspear 2012; Farina et al. 2013; Ward et al. 2013) as well as previous studies on intermuscular coherence during quiet standing (Danna-Dos-Santos et al. 2014, 2015; Obata et al. 2014).

The procedure used for the EMG–EMG coherence analysis was similar to those reported previously (Poston et al. 2010; Danna-Dos-Santos et al. 2015). The functional synergies related to the anterior and posterior M-modes described above were tested in this study (Table 1). Common neural inputs in the core muscles were also established. Moreover, antagonist synergies of calf and thigh muscles were calculated. Finally, coherence was computed between muscles with no synergistic relation (mixed group in Table 1). The latter muscle pair group performed the role of a control group. Including all these muscle pairs thus allowed us to determine the existence of: (1) correlated neural commands in the anterior muscles, (2) correlated neural commands in the posterior muscles, (3) correlated neural commands in the core muscles and (4) correlated neural commands in antagonist leg muscles.

**Table 1:** Muscle pairs used during intermuscular coherence analyses (pooled and single pair)

	Muscle pair	Muscular synergy
1	GM-BF	Posterior
2	GM-ES	Posterior
3	BF-ES	Posterior
4	TA-VM	Anterior
5	TA-RA	Anterior
6	VM-RA	Anterior
7	RA-ES	Core
8	RA-EO	Core
9	ES-EO	Core
10	GM-VM	Mixed
11	GM-RA	Mixed
12	VM-ES	Mixed
13	TA-BF	Mixed
14	TA-ES	Mixed
15	BF-RA	Mixed
16	TA-GM	Antagonist
17	VM-BF	Antagonist

*TA* tibialis anterior, *GM* gastrocnemius medialis, *VM* vastus medialis, *BF* biceps femoris, *RA* rectus abdominis, *EO* external oblique, *ES* erector spinae

Single-pair coherence estimations were performed by dividing the squared cross-spectrum of two EMG signals by the product of the auto-spectrum of each signal:

1 $$\left| {R_{xy} \left( \lambda \right)} \right|^{2} = \frac{{\left| {f_{xy} \left( \lambda \right)} \right|^{2} }}{{\left| {f_{xx} \left( \lambda \right)f_{yy} \left( \lambda \right)} \right|}}$$

where $R_{xy} \left( \lambda \right)$ is the intermuscular coherence, $f_{xy} \left( \lambda \right)$ is the cross-spectrum between both EMG signals, $f_{xx} \left( \lambda \right)$ is the auto-spectrum of the first EMG signal and $f_{yy} \left( \lambda \right)$ is the auto-spectrum of the second EMG signal.

The estimated coherence was obtained using the Welch method with a non-overlapping Hanning window of 1024 points (frequency resolution = 0.98 Hz). The range of the frequencies analysed in this study varied from 0 to 55 Hz. The coherence confidence limit was set at 0.0739, following the equation proposed by Rosenberg et al. (1989).

Coherence estimations were normalized (Fisher transformation) to perform comparisons between the two experimental conditions. The coherence signal was an integer over the range interval between 0 and 5 Hz (i.e. common drive) because significant coherence was found only in that frequency band (Poston et al. 2010).

Five pooled coherence estimations were also quantified. The first included the three pairs of anterior muscles (TA/VM, TA/RA and VM/RA), the second the pairs of posterior muscles (GM/BF, GM/ES and BF/ES), the third the pairs of core muscles (RA/ES, RA/EO and ES/EO), the fourth the mixed pairs (Table 1) and the last the antagonist pairs (TA/GM and VM/BF). The pooled coherence was computed as the weighted average of individual coherence estimation pairs (Poston et al. 2010; Danna-Dos-Santos et al. 2014, 2015; Obata et al. 2014). The pooled coherence was normalized using Fisher transformation and integer in the same frequency interval as single coherence.

### Statistical analysis

Statistical analysis was performed with SPSS 20 (IBM, Armonk, USA). The normality (Shapiro–Wilk test) assumption was checked, and as several variables did not meet this assumption, nonparametric tests were applied (supplementary material). Descriptive methods were used to compute the median and the interquartile range. Wilcoxon signed-rank tests were applied to establish differences between conditions (i.e. bipedal and unipedal) in CoP variables and mean RMS electromyography. Friedman’s ANOVA was applied to establish differences between groups of muscle pairs (i.e. anterior, posterior, core, mixed and antagonist groups) as regards pooled coherence integral values. Friedman’s ANOVA was applied to establish differences in the single coherence between pairs of muscles that form the same synergy. The follow-up to Friedman’s ANOVA was carried out by means of multiple Wilcoxon signed-rank tests. Due to the multiple comparisons, a Bonferroni correction was used. Finally, differences in the pooled and single coherence between conditions (i.e. bipedal and unipedal) were established by means of the Wilcoxon signed-rank test. The level of significance was set at *p* = 0.05.

## Results

### CoP variables

Significant differences in all the CoP variables were found between bipedal and unipedal stance conditions. In fact, all the variables presented a higher median value in the unipedal than the bipedal stance condition. Table 2 shows the descriptive statistics as well as Wilcoxon signed-rank test statistic, effect size and *p* values.

**Table 2:** Differences between conditions in CoP variables

	Direction	Bipedal	Unipedal	*z*	*r*	*p* value
EA (mm^2^)	–	114.60 (170.09)	628.23 (307.99)	−3.88	−0.61	<0.001
RMS (mm)	ML	2.19 (2.10)	5.35 (1.15)	−3.92	−0.62	<0.001
	AP	3.46 (2.19)	6.62 (2.49)	−2.84	−0.45	0.005
MV (mm s^−1^)	ML	3.53 (2.12)	24.52 (17.01)	−3.92	−0.62	<0.001
	AP	5.02 (2.35)	22.74 (12.88)	−3.92	−0.62	<0.001
F80 (Hz)	ML	0.80 (0.41)	1.12 (0.49)	−3.3	−0.52	0.001
	AP	0.59 (0.47)	1.05 (0.41)	−3.92	−0.62	<0.001
F50 (Hz)	ML	0.54 (0.21)	0.67 (0.30)	−2.24	−0.35	0.025
	AP	0.32 (0.16)	0.67 (0.34)	−3.87	−0.61	<0.001

Data are expressed as median (interquartile range)

*EA* ellipse area, *RMS* root mean square, *MV* mean velocity, *F80* 80 % energy frequency, *F50* 50 % energy frequency, *AP* anteroposterior, *ML* mediolateral

### EMG time domain variables

**Fig. 2:**
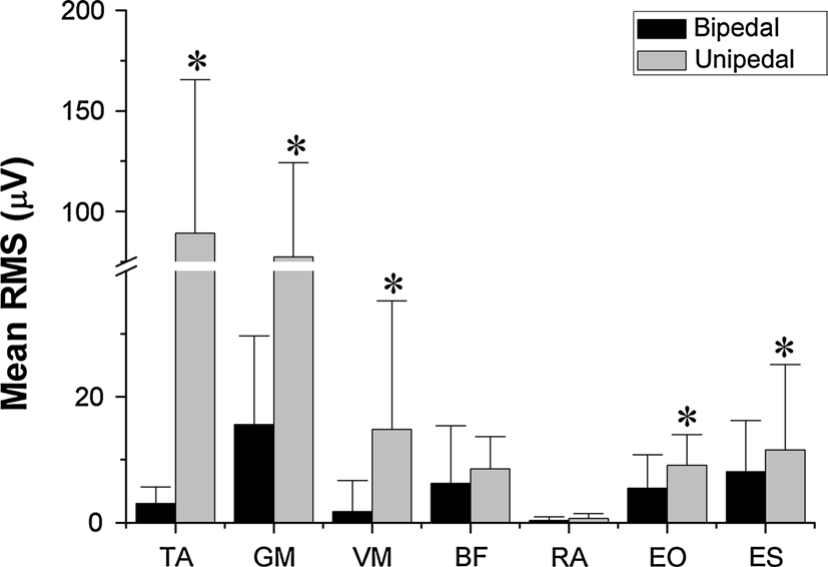
Mean electromyography RMS comparison between different conditions. *Bars* represent the median value and* error bars* the interquartile range. *Asterisk* indicates significant differences related to bipedal condition (*p* < 0.05). *TA* tibialis anterior, *GM* gastrocnemius medialis, *VM* vastus medialis, *BF* biceps femoris, *RA* rectus abdominis, *EO* external oblique, *ES* erector spinae

Significant differences in the mean RMS between conditions were observed (Fig. 2). The mean RMS was higher in the unipedal stance condition in TA (*z* = −3.92; *p* < 0.001; *r* = −0.62), GM (*z* = −3.92; *p* < 0.001; *r* = −0.62), VM (*z* = −3.66; *p* < 0.001; *r* = −0.58), EO (*z* = −2.54; *p* = 0.01; *r* = −0.4) and ES (*z* = −3.21; *p* = 0.001; *z* = 0.51).

### Pooled coherence

**Fig. 3:**
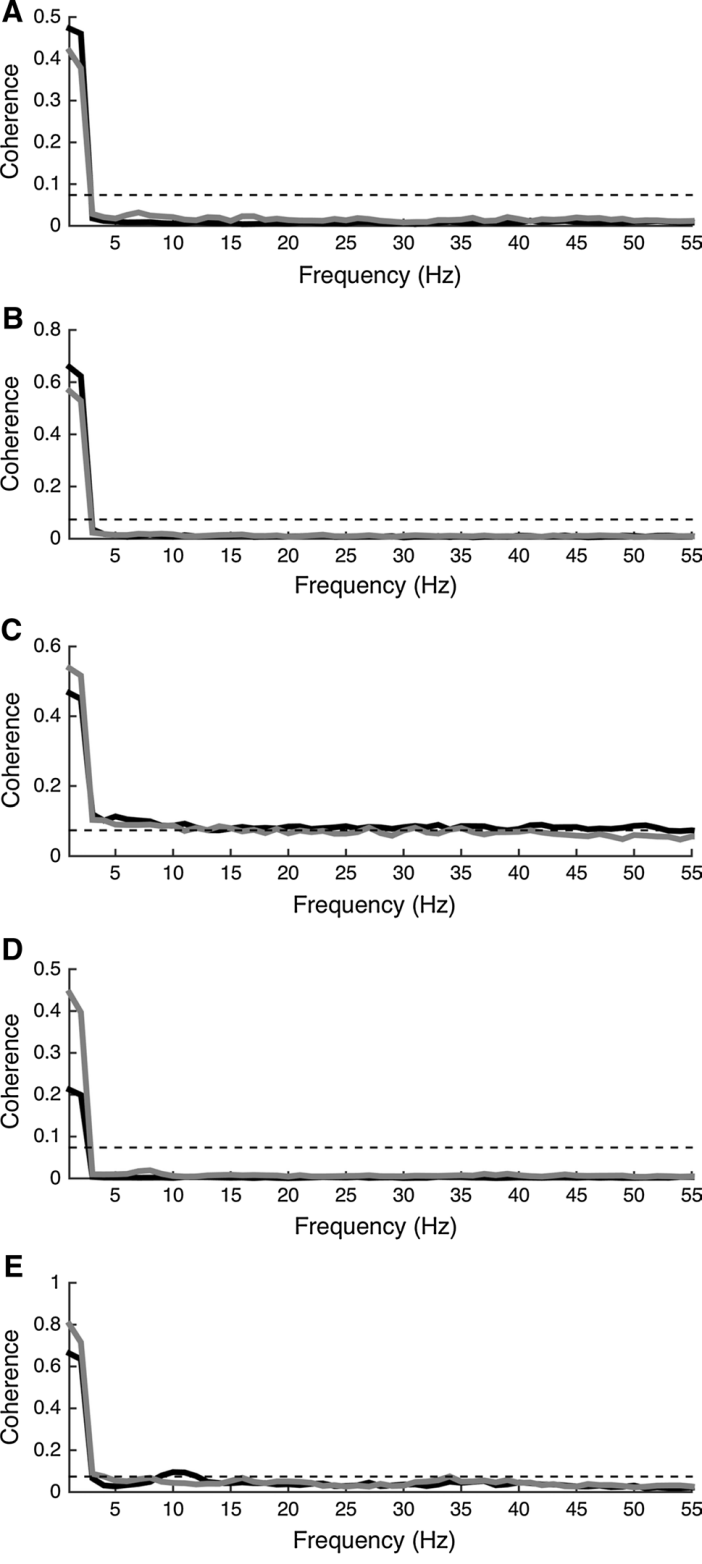
EMG-EMG pooled coherence. **a** Pooled coherence for anterior M-mode muscles.** b** Pooled coherence for posterior M-mode muscles.** c** Pooled coherence for core muscles.** d** Pooled coherence for mixed muscles group. **e** Pooled coherence for the antagonist muscle group. The coherence of bipedal stance condition is marked in *black*, while the *dark grey line* relates to the coherence during unipedal stance condition. The *dotted line* represents the level of significance for coherence analysis (i.e. 0.0739)

As the pooled coherence of anterior, posterior, core, mixed pairs of muscles and antagonist muscles was significant in the 0–5 Hz frequency band (Fig. 3), the integral value of the frequency bands was compared between these muscles groups as well as between the conditions. It is important to note that the coherence of core muscles reached the significance level throughout almost all the frequency range.

**Fig. 4:**
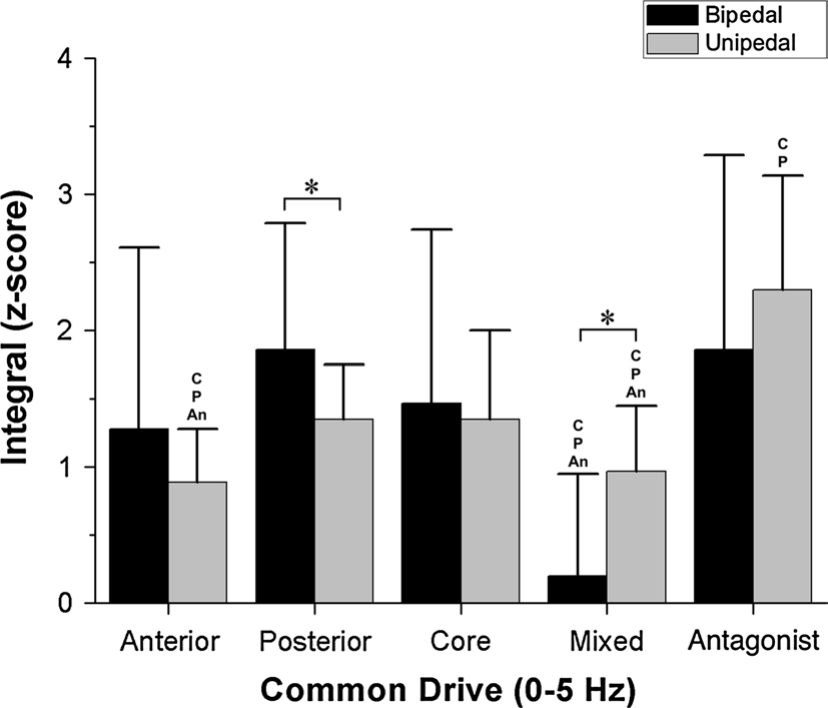
Comparison between muscle groups and conditions versus the integral of the normalized coherence between 0 and 5 Hz. The bars represent the median and the* error bars* the interquartile range. *Asterisk* indicates the significant differences between bipedal and unipedal conditions. *An* refers to significant differences related to the antagonist M-mode. *P* indicates significant differences related to posterior M-mode. *C* relates to significant differences of the core muscles

Friedman’s ANOVA revealed a significant effect of the group on the common drive band ($\chi_{4}^{2}$ = 23.88; *p* < 0.001) in the bipedal stance condition. The pairwise comparisons are shown in Fig. 4. In this band, the mixed group showed a lower integral than the posterior, core and antagonist groups.

In the unipedal stance, Friedman’s ANOVA showed an effect of the muscle group in common drive ($\chi_{4}^{2}$ = 54.2; *p* < 0.001). In this condition, the mixed and anterior groups showed lower values than the posterior, core and antagonist muscle groups (Fig. 4). Moreover, the antagonist integral value was larger than the integral values of core and posterior groups.

A comparison of the conditions (Fig. 4) shows that the mixed group integral value was higher in unipedal than in bipedal stance (*z* = −3.43; *p* = 0.001; *r* = −0.54). Finally, the posterior muscle group integral value was higher in bipedal than in unipedal stance in the common drive frequency band (*z* = −2.35; *p* = 0.019; *r* = −0.37).

### Single coherence

As the coherence of single muscle pairs reached values above the threshold confidence level in the 0–5 Hz frequency band, the integral values were computed in the same way as the pooled coherence values.

No differences between the integral values of the pairs of muscles involved in the same M-mode in the bipedal stance were found. Nevertheless, significant differences between TA/GM and VM/BF integral value in bipedal condition (*z* = −2.58; *p* = 0,01; *r* = 0.41) were observed. Regarding unipedal stance, the muscle pair did have an effect on the integral value of the anterior M-mode ($\chi_{2}^{2}$ = 15.7; *p* < 0.001) and core muscles ($\chi_{2}^{2}$ = 24.7; *p* < 0.001). Pairwise comparisons revealed that the TA/VM integral was lower than VM/RA (*z* = −3.95; *p* < 0.001; *r* = −0.62), while the EO/ES integral was smaller than the RA/EO and RA/ES integrals (*z* = 4.9; *p* < 0.001; *r* = 0.77 and *z* = 3.16; *p* = 0.005; *r* = 0.5, respectively).

Comparisons between the integral values of both conditions showed several significant dissimilarities (Table 3). In the anterior muscle pairs, differences in TA/VM and TA/RA coherence between the conditions were observed. The integral values were higher in the bipedal than unipedal stance. No differences were found between conditions in any posterior muscle pair. Regarding the core muscle pairs, EO/ES coherence presented a higher integral value in the bipedal than in unipedal stance. Significant differences in mixed muscle pairs were observed. TA/BF and TA/ES revealed higher integral values in the bipedal than in unipedal stance. Finally, the coherence of TA/GM was higher in bipedal stance than in unipedal stance.

**Table 3:** Integral coherence value of single pairs of muscles

	Bipedal stance	Unipedal stance
TA/VM	4.29 (2.50)*	2.45 (1.08)
TA/RA	4.48 (1.05)*	2.61 (0.94)
VM/RA	4.53 (1.69)	3.57 (1.81)
GM/BF	3.52 (1.56)	2.78 (1.34)
GM/ES	3.73 (1.42)	3.65 (1.02)
BF/ES	3.23 (2.10)	2.88 (1.33)
RA/EO	8.24 (6.28)	9.44 (8.81)
RA/ES	5.04 (6.32)	4.70 (2.32)
EO/ES	4.58 (9.01)*	4.22 (1.02)
GM/VM	3.59 (1.51)	3.17 (1.54)
GM/RA	3.95 (0.91)	3.94 (0.51)
VM/ES	3.91 (2.38)	2.92 (1.73)
TA/BF	4.16 (2.34)*	2.04 (1.24)
TA/ES	4.25 (1.33)*	2.18 (0.74)
BF/RA	3.60 (2.33)	3.00 (1.44)
TA-GM	4.93 (1.24)*	2.56 (0.78)
VM-BF	2.52 (3.24)	3.15 (1.99)

Data are expressed as median (interquartile range)

*TA* tibialis anterior, *GM* gastrocnemius medialis, *VM* vastus medialis, *BF* biceps femoris, *RA* rectus abdominis, *EO* external oblique, *ES* erector spinae

* Indicates significant differences between conditions (*p* < 0.05)

## Discussion

In our study, we have found significant coherences in the 0–5 Hz frequency band in the anterior and posterior M-modes, core muscles and antagonist muscles of the leg during the balance tests. It was also observed that in the groups formed by the anterior (not significant), posterior, core and antagonist muscles the coherence integral was greater than the mixed muscles in the bipedal condition (in theory not synergistic). This confirms the existence of common neural inputs to the muscles to create muscle synergies for maintaining balance, which has also been observed in the posterior and anterior muscles in other studies (Danna-Dos-Santos et al. 2014, 2015). However, up to date there have been no studies examining the core muscles during the static balance task. In addition, ours is the first study that analyses EMG coherence among the different muscle groups while performing single-leg static balance.

In the wide field of motor control and, particularly, in the field of static postural control, the study of motor variability derived from the redundant degrees of freedom and muscle actions is of great importance (Latash et al. 2002). The CNS should be able to develop appropriate strategies to solve problems, and these responses will be implemented by a system with multiple degrees of freedom. Some authors have proposed to simplify the problem by considering that the CNS organizes groups of structural elements or units with a common goal (Gelfand and Tsetlin 1966). In this way, the CNS seems to produce patterns of general operations that are sent to the structural units. If an error occurs in the execution of some of the elements that form a structural unit, other elements might compensate for the error. Systems operating according to these principles are considered to be synergies.

Considering the uncontrolled manifold (UCM) hypothesis, it was found that control of bipedal upright posture while performing voluntary sway or tasks that required arm movements was conducted by two muscular synergies (Krishnamoorthy et al. 2003) composed of an anterior and a posterior synergy. The anterior muscle synergy is responsible for moving the centre of mass forward, while the posterior acts in the opposite direction.

Although these synergies have been detected using the UCM hypothesis, it is still not known in depth how the CNS controls these structural units. Some authors suggest that it sends correlated neural inputs to the different muscles involved in each synergy, a hypothesis that has been checked in previous studies (Danna-Dos-Santos et al. 2014, 2015). In addition, it has been observed that when the sensorial visual inputs are removed, the correlated neural inputs diminish (Danna-Dos-Santos et al. 2015). Therefore, a deficit of visual information may imply that the CNS does not use the same muscular synergies, since these are task-specific synergies.

In the present study, we found that there are common neural inputs at low-frequency oscillations (i.e. between 0 and 5 Hz) in the muscles forming the anterior and posterior M-modes during the performance of static bipedal balance tasks. Previous studies delivered small differences in the frequency band in which significant coherence was found. For example, Danna-Dos-Santos et al. reported significant coherences between 0 and 20 Hz in the anterior M-mode in a “holding load forward” condition (Danna-Dos-Santos et al. 2014). However, the results of a recent paper show significant coherence only in the 0–10 Hz frequency band (Danna-Dos-Santos et al. 2015). Obata et al. compared the coherence integral in children, adults and the elderly, reporting significant coherence in the 0–4 Hz band in all three groups, as well as between 8 and 12 Hz in the older group (Obata et al. 2014). Only young adults were used in the present study, and our results are very similar to those obtained by Obata et al. (2014) regarding significant frequency bands.

This frequency band is related to a modulation known as the “common drive”, which has a central origin (Kamen and De Luca 1992), but it is still not known in which centre it is generated. As it has been proven that people who suffered a cortical or capsular stroke keep the common drive (Farmer et al. 1993), these centres cannot be responsible for its generation. Oscillations in this frequency are produced in movements requiring constant or slowly increasing forces (Farmer et al. 1993; De Luca and Erim 1994). In this context, the fact that we found significant coherence values in the common drive frequency seems to be plausible, since it matches with the former description.

Although anterior and posterior muscles synergies were found by Krishnamoorthy et al. (2003), another study found more M-modes (without observing synergy) during the performance of different postural control tasks (Krishnamoorthy et al. 2004). It was hypothesized that the CNS chooses some M-modes from a task dependent “menu” of M-modes to maintain postural balance (Krishnamoorthy et al. 2004). In this study, reciprocal and co-contraction M-modes were found.

In this sense, our study reveals a large integral value of the spectral coherence in antagonist muscles and in core muscles. Therefore, this coherence could explain the M-modes co-contraction found in a previous study (Krishnamoorthy et al. 2004). Antagonist co-contraction has been considered efficient enough to increase joint stiffness and therefore to stabilize the joint during the performance of motor tasks such as upright posture (Hansen et al. 2002; Geertsen et al. 2013). Moreover, ankle joint stiffness has been accepted as one of the mechanisms involved in keeping balance in control studies (Baratto et al. 2002).

Regarding core muscles coherence, previous studies suggest that these muscles could provide the main muscle synergy for spine stability (Key 2013). In this regard, we found that the integral of the spectral coherence between these muscle groups was similar in both the anterior and posterior groups for the 0–5 Hz band. Furthermore, it is also possible that these muscles share neural inputs in other frequency bands, as we found values very close to the significance threshold in the rest of the analysed band width values.

However, it seems that the most plausible explanation is that the core muscles are compensating for inspiratory and expiratory movements that occur during breathing (Hodges et al. 2002; David et al. 2015). Although our study does not allow us to corroborate this hypothesis (i.e. we did not measure the respiratory rate in our subjects), it seems that the coherence we found between the different core muscles at lower frequencies points towards this type of CNS strategy to compensate for trunk movements due to breathing.

Of course, greater control of core muscles could also improve head stability, since these muscles control the movements of the lumbar vertebrae and the hips, which make a fundamental contribution to the width of movement in this body segment (Peterson et al. 2001).

Regarding the unipedal stance condition, it was found that the integral value was higher in posterior, core and antagonist muscles than in mixed and anterior muscles. This could mean that anterior synergies are not created during static unipedal balance, so that it would be interesting to use the UCM hypothesis to determine the actual muscles synergies in play during the single-leg stance task. Moreover, the integral value was lower in the unipedal than the bipedal stance condition in anterior (non-significant), posterior (significant) and core (non-significant) muscles, although the integral value for mixed and antagonist muscles was higher in the unipedal stance condition. This new landscape in EMG–EMG coherence during the unipedal stance test confirms that the synergies are task dependent (Latash et al. 2002).

Finally, stability and control parameters, such as the ellipse area covered and the mean velocity of the CoP, were significantly higher in unipedal stance. In addition, RMS EMG was larger in unipedal stance for the TA, GM, VM, EO and ES muscles. This increase in muscle activation (and therefore muscle force production) led to modifications in the common neural inputs of the different muscle pairs tested. This contradicts the results reported by Poston et al. during different force production in grasping tasks (Poston et al. 2010). However, not only force production but also muscle coordination could be different in unipedal and bipedal stance conditions.

Obviously, our work has some limitations that should be taken into account. Body segment position changes involved in balance control were not measured, since we could not perform kinematic analysis in our laboratory. This fact limits the conclusions of this study. Moreover, EMG recordings of both lower limbs during bipedal and unipedal stance tasks would provide additional information. This is important because for bipedal stance both lower limbs are working together, while the dominant limb acts alone in the unipedal stance test.

On the other hand, not having included a large number of trials restricted the type of analysis we could perform. Future research should include a larger number of trials for each condition in order to elucidate the synergies produced through analytic methods based on UCM hypothesis, such as principal component analysis (Krishnamoorthy et al. 2003). To corroborate our results, this type of experimental design should be carried out together with a high number of trials including the core muscles.

Even though the main findings of our study could be regarded as theoretical, the practical applications point out to the importance of adopting strategies involving core training for improving balance in persons with pathologies entailing problems in balance control (Park and Hwangbo 2014).

Our main conclusion is that the core and antagonist muscle groups, such as the anterior and posterior muscles, share low-frequency neural inputs (0–5 Hz). These common inputs might be responsible of the assembly of the M-modes. In this sense, our results support the hypothesis that the CNS is dictating the M-modes conformation and therefore responsible for postural control handling with the additional problem of high degrees of freedom. However, our results do not permit us to identify exactly where the process takes place: either at the supraspinal or at spinal medulla levels of the nervous system. In addition, we found important differences between the unipedal and bipedal stance conditions regarding postural control and stability, as well as in muscle activation. It is thus possible that the muscle synergies involved in unipedal stance tasks are different to those required for the bipedal stance condition.

## Electronic supplementary material

Below is the link to the electronic supplementary material.

Supplementary material 1 (XLSX 35 kb)
